# Vigabatrin (Sabril) for the Treatment of Refractory Complex Focal Seizures in Adults: Pharmacologic and Clinical Considerations

**DOI:** 10.7759/cureus.46414

**Published:** 2023-10-03

**Authors:** Taylor N Gatson, Orlandria A Freemont, Rachel L Coleman, Stewart J Lockett, Noah J Spillers, Shahab Ahmadzadeh, Omar Viswanath, Giustino Varrassi, Sahar Shekoohi, Alan D Kaye

**Affiliations:** 1 Medicine, Louisiana State University Health Sciences Center at Shreveport, Shreveport, USA; 2 Anesthesiology, Louisiana State University Health Sciences Center at Shreveport, Shreveport, USA; 3 Pain Management, Valley Pain Consultants - Envision Physician Services, Phoenix, USA; 4 Pain Medicine, Paolo Procacci Foundation, Rome, ITA

**Keywords:** seizure, vigabatrin, epilepsy, refractory complex partial seizures, sabril

## Abstract

To review the pharmacokinetics, efficacy, and adverse effects of vigabatrin (Sabril) and its role in managing refractory focal unaware seizures in adults. In the present investigation, a search of English-language literature from 1999 through 2023 was conducted using vigabatrin and Sabril as search terms to identify relevant studies and review articles. A 2000 double-blind, placebo-controlled multicenter study found that out of 90 adult patients, 48% of those treated with vigabatrin achieved a 50% or greater reduction in the frequency of complex partial seizures, compared to 26% of placebo-treated patients. This study also observed that vigabatrin was well tolerated by 72.4% of patients, with the most common side effects being drowsiness, dizziness, headache, and fatigue. Further studies with a significant risk of vigabatrin-associated visual field loss necessitate vigabatrin only being an option for refractory cases. Additional studies suggest that despite the potential risk of vision loss and adverse effects, adult patients continue to use vigabatrin long-term. Sabril is an antiepileptic medication prescribed as an additional treatment for refractory complex partial seizures in patients at least ten years old who have not responded well to other alternative therapies. Multiple clinical trials indicate that Sabril sufficiently reduces the frequency of seizures when used as an adjunct treatment of refractory complex focal seizures. However, it is important to carefully monitor patients for any adverse effects, particularly long-term use, and to discontinue the drug if serious side effects occur.

## Introduction and background

Epilepsy is the second most prevalent neurologic disorder globally, affecting over 50 million people who experience repeated unprovoked seizures [1]. In the United States, approximately 3 million individuals of various age groups have seizure disorders, and about 200,000 new cases of seizures and epilepsy are diagnosed annually [2]. In the world, approximately 50 million people have epilepsy [3-10]. The total cost of epilepsy in the world is estimated to be close to 119 billion dollars [11-18]. Essentially, epilepsy can be classified into two main categories: primary generalized epilepsy and focal seizures. The latter is characterized by four types of seizures - focal with impaired awareness, focal with awareness, focal tonic-clonic, and bilateral tonic-clonic. The clinical diagnosis of complex partial epilepsy relies on a combination of the patient's clinical history, laboratory tests, and neuroimaging studies. Roughly half of patients with this type of seizure disorder can attain complete control over their epilepsy symptoms with medication. Still, the other half either continue to experience seizures despite being on anti-seizure medications or suffer significant side effects in pursuit of complete seizure control [19]. When two or more antiepileptic drugs fail to manage the seizures, they are referred to as refractory. Based on these facts, it can be inferred that more than 7.5 million people worldwide suffer from refractory complex partial seizures [19,20]. The primary objective of epilepsy management is to eliminate seizures in patients, including those with refractory complex partial seizures, without inducing drug-related side effects. This can often be achieved by selecting the most appropriate anti-seizure medication, a combination of anti-seizure medications, or surgical removal of the seizure focus. In the United States, there are currently four traditional first-line anti-seizure medications (phenobarbital, phenytoin, carbamazepine, valproate) and 13 newer anti-seizure medications (felbamate, gabapentin, lamotrigine, topiramate, tiagabine, levetiracetam, oxcarbazepine, zonisamide, pregabalin, lacosamide, vigabatrin, perampanel, and cenobomate) available for treating localization-related epilepsy [2,19]. However, current research shows no anti-seizure medication is superior to others for managing refractory complex partial seizures [20,21]. Hence, when choosing the appropriate anti-seizure for an individual patient, other factors must be considered, including the potential adverse reactions that the patient may experience.

Vigabatrin (brand name: Sabril®) is an anti-seizure medication prescribed as an additional treatment for refractory complex partial seizures in patients aged ≥ 10 years who do not respond well to alternative therapies. Vigabatrin is also used as monotherapy to treat infantile spasms; however, this study aimed to evaluate vigabatrin's effectiveness in treating adults. This medication is a structural derivative of γ-aminobutyric acid (GABA), the primary inhibitory neurotransmitter in the central nervous system (CNS). It is available in over 60 countries globally and received Food and Drug Administration (FDA) approval in the United States in 2009 [2]. As standard antiepileptic drugs (AEDs) fail to control seizures in about one-third of patients with epilepsy, and the newer agents only benefit less than half of those individuals, vigabatrin may be a viable option for treating refractory epilepsy. However, the advantages of using vigabatrin must be weighed against its potential risks, with the most significant risk being the loss of peripheral vision [19]. Assessing the risks associated with vigabatrin is crucial in light of its application. Epilepsy is a severe medical condition, and uncontrolled seizures can cause substantial disability or even lead to death [22,23]. The present investigation, therefore, focused on the role of Sabril as adjunctive therapy in managing refractory complex focal seizures in adult patients. This review aims to examine the effectiveness, safety, and potential adverse effects of Sabril.

## Review

Methods 

In the present investigation, a search of English-language literature from 1999 through 2023 was conducted using vigabatrin and Sabril as search terms to identify relevant studies and review articles. This was a narrative review. The sources for this review are as follows: searching on PubMed, Google Scholar, Medline, and ScienceDirect; using keywords: 'Sabril', 'refractory complex partial seizures', 'epilepsy', 'vigabatrin'. Sources were accessed between February 2023 and May 2023. Investigators were instructed to focus their study on the relevant keywords. The focus group refrained from reviewing vigabatrin’s use in children. To ensure the reliability and validity of the included studies, the research team critically appraised the methodological quality of each selected article. This assessment evaluated the study design, sample size, and data collection methods. The team synthesized the extracted data and summarized the key findings. Collected findings were tabulated according to the respective category. Data was organized thematically or by relevant subtopics to facilitate a comprehensive analysis.

Pharmacokinetics of vigabatrin 

Vigabatrin is used as an antiepileptic to treat refractory complex seizures. Vigabatrin works by indirectly increasing levels of the neurotransmitter GABA. GABA is an amino acid that serves as an inhibitory neurotransmitter in the brain [23]. The current belief is that seizures are caused by low levels of GABA in the central nervous system [2]. Vigabatrin works by irreversibly inhibiting gamma-aminobutyric acid transaminase (GABAT-T). This enzyme functions to degrade GABA in the synapse. By inhibiting GABA-T, vigabatrin increases the amount of GABA in the synapse, thus terminating seizure activity. Vigabatrin has also been shown to directly prevent the uptake of and stimulate the release of GABA into the neuronal synapse. It has been shown that vigabatrin enhances the action of glutamine. Glutamine is a precursor of GABA, so enhancing glutamine would increase GABA in the synapse, adding to the anticonvulsant effect [23].

Vigabatrin is administered orally in tablet or powder form and is absorbed completely orally. The starting dose in adults is 500 mg, twice a day (BID) and may be titrated weekly to a maximum dose of 3 grams, administered as 1500 mg BID. Vigabatrin does not bind to plasma proteins and, as a result, is widely distributed. The medication takes about one hour to achieve peak plasma concentration in adults and two and a half hours in infants. The drug's availability in the body depends on the rate of enzyme re-synthesis, not the rate of elimination of the drug from circulation. No plasma concentration monitoring is required with the administration of the drug [22,23]. Vigabatrin is almost entirely excreted by the kidney (about 80-95%) and has a half-life of about 7-7.5 hours in adults. The half-life is approximately 5.5 hours in infants. In patients with renal impairment, the dose should be adjusted in relation to the creatinine clearance. Vigabatrin is considered an inducer of cytochrome P450 2CP but does not undergo significant hepatic metabolism, so no adjustments or monitoring are required in patients with liver failure. Vigabatrin has not been shown to interact with steroid contraceptives. Vigabatrin does not cause a significant decrease in concentration when co-administered with other anti-seizure medications, with the exception of phenytoin, whose total plasma levels are decreased when taken with Vigabatrin. There may be a need for adjustments when co-administering the two medications [2]. This drug should be used with caution in patients who are pregnant or breastfeeding; it is classified as a category C medication in pregnancy. In mice studies, there has been some association with Vigabatrin causing fetal loss, intrauterine growth restriction, and alterations in folate and B12 [21]. Vigabatrin should also be used with caution in women who are breastfeeding. Vigabatrin toxicity is not common but may occur gradually after prolonged treatment. There are many associated nonspecific symptoms of vigabatrin toxicity, including drowsiness, unconsciousness, agitation, headache, irritability, psychosis, bradycardia, status epilepticus, and even coma. Most patients recover with supportive care and symptomatic treatment. In severe overdose, diazepam and haloperidol have been shown to be useful. Because vigabatrin is renally excreted, hemodialysis could treat overdose and reduce drug concentration by 40-60 percent. For patients with renal impairment, the dose should be adjusted based on CrCl. There are no absolute contraindications for the use of vigabatrin, but the drug does have some adverse side effects and a black-boxed warning [23].

Side effects of vigabatrin and contraindications in adult population 

Vigabatrin is generally a well-tolerated pharmaceutical drug. In the 36-week double-blind, no changes were observed in the pulse, respiration rate, or blood pressure (systolic and diastolic) [2]. clinical trials show that vigabatrin's most common side effects are headache and drowsiness. Although commonly found throughout several clinical trials of vigabatrin, the severity of these symptoms did not indicate discontinuation of the trials [19]. Drowsiness and headaches remain consistent side effects of many anti-seizure medications and are generally not debilitating and are self-limiting. In 20% to 30% of patients treated with vigabatrin, MRI hyperintensities were noted in the basal ganglia, thalami, and brainstem on diffusion-weighted and T2/fluid-attenuated inversion recovery (FLAIR) sequences. These findings were insignificant and disappeared when vigabatrin was discontinued [22,24]. Other side effects include convulsions, upper respiratory tract infections, nasopharyngitis, nystagmus, tremors, blurred vision, memory impairment, weight gain, arthralgia, abnormal coordination, nausea, and diarrhea. These side effects also did not warrant discontinuation of any trials [2]. There were two isolated instances of acute psychosis that caused discontinuation in St. Michael’s with one patient having a previous diagnosis of schizophrenia [25].

The most severe side effect is peripheral vision loss, with a black box warning that over 30% of adults will experience some form of peripheral vision loss [2]. Trials have shown that peripheral visual defects (VFDs) occur bilaterally as early as the nine-month mark in adults and on average around the ⅚ year mark, while central vision remains unchanged [23]. Related to this potential side effect, the Food and Drug Administration (FDA has mandated a recommendation that anyone starting vigabatrin should get a baseline ophthalmological exam [26].

Teratogenicity was studied in a research study involving pregnant mice. The mice were injected with a “high dose” (450mg/kg) of vigabatrin and a “low dose” (350 mg/kg). The results showed fetal demise in the high dose and severe intrauterine restrictions in the low dose [27]. Additionally, both groups had a 50% decrease in Folate and B12 levels [27]. These trials indicate that pregnancy may be a direct contraindication to vigabatrin. Vigabatrin is commonly co-administered with other anti-seizure medications. Within multi-drug related trials, it was shown that vigabatrin is a GABA transaminase and is thus suspected of also being an inhibitor of alanine and aspartate aminotransferase, which has been shown to lower slightly elevated liver enzymes to a therapeutic range. This was shown to be reversible by mixing vigabatrin with plasma. In the preclinical trials, there was no evidence of elevated liver enzymes or apparent liver injury [28]. Longitudinal follow-up has linked vigabatrin to isolated cases of liver failure suspected to be by hypersensitivity and not direct hepatotoxicity because the drug is metabolized by many tissues in the body. Although the suspected mechanism of action (MOA) is hypersensitivity, one case study showed the possibility that vigabatrin may be the cause of vigabatrin-induced liver injury after standardizing for autoimmune and hypersensitivity possibilities [28]. To note, side effects were not found to affect a particular demographic and were seen consistently across the board throughout all trials. Although vigabatrin is not without side effects, it remains a crucial adjunct treatment for refractory complex focal seizures in adults as the benefits outweigh the risks. 

Efficacy of treatment with Sabril 

Multiple studies have been conducted to investigate the efficacy of vigabatrin for the adjunct treatment of refractory complex focal seizures prior to and following its FDA approval in 2009. Furthermore, the prevalence of side effects, most notably vision loss, has also been studied. A 2000 double-blind, placebo-controlled multicenter study investigated the efficacy and tolerability of vigabatrin as an adjunctive treatment of partial seizures over a 36-week titration and maintenance phase. The study found that out of 90 adult patients, 48% of those treated with vigabatrin achieved a 50% or greater reduction in the frequency of secondarily generalized partial seizures and complex partial seizures, compared to 26% of placebo-treated patients. It was also observed that vigabatrin was well tolerated by 72.4% of patients, with the most common side effects being drowsiness, dizziness, headache, and fatigue. 12.1% of vigabatrin-treated patients and 7.6% of placebo patients poorly or very poorly tolerated vigabatrin (Table 1). Overall, these data suggest that vigabatrin was well-tolerated and effective adjunctive treatment of refractory complex partial seizures and secondarily generalized partial seizures [25].

**Table 1:** Clinical efficacy and safety

Author (Year)	Groups studied and intervention	Results and findings	Conclusions
Cruz et al. [2]	174 adults (18-60 y.o.) with complex partial seizures with or without secondary generalization who failed a phenytoin or carbamazepine regimen were given placebo or vigabatrin 1g/day (n=45), 3 g/day (n=41), or 6 g/day (n=43). Seizure events recorded during 8-week baseline period and 18-week treatment period.	51% of patients given vigabatrin 6 g/day, 53% of patients given 3 g/day, and 9% of patients given placebo reached a 50% or greater reduction in seizure frequency.	Vigabatrin significantly reduced the frequency of seizures when used as an adjunct treatment of refractory complex focal seizures.
Cruz et al. [2]	182 adults (18-60 y.o.) with complex partial seizures with or without secondary generalization were randomly treated with placebo (n=90) and vigabatrin (n=92) 3 g/day, and their median monthly frequency of complex partial seizures were assessed over an 8-week baseline period and 16-week treatment period.	~39% of vigabatrin treated patients and 21% of placebo treated patients reached a 50% or greater reduction in seizure frequency.	Vigabatrin reduced the median monthly frequency of complex partial seizures in patients treated with vigabatrin.
Krauss et al. [19]	Adult patients (age 17 years or older) with refractory complex seizures who receive vigabatrin as adjunctive therapy and are registered in the Sabril® registry.	In patients naïve to vigabatrin, 71% remained in the registry at 3 months, 55% at 6 months, and 40% at 12 months.	Despite having refractory epilepsy and despite the risks of adverse effects, a significant proportion of adult patients remain vigabatrin
Bresnahan et al. [25]	Adult patients (16-50 y.o.) in different cities in Canada in 1991-1992 with complex partial seizures or partial seizures with secondary generalization were randomized to be given either vigabatrin or a placebo for a 36-week treatment duration. Efficacy was assessed by recording the number and types of seizures the patient experienced.	A 50% or greater reduction in the frequency of seizures was seen in 48% of patients treated with vigabatrin and in 26% of patients treated with placebo. Only 10% of patients treated with vigabatrin discontinued treatment early due to adverse effects, compared to 8% of placebo patients. 72.4% of vigabatrin treated patients reported tolerating vigabatrin well, 12.1% of vigabatrin patients, and 7.6% of placebo patients poorly or very poorly tolerated vigabatrin.	Vigabatrin is well tolerated and effective in treating complex partial and tonic-clonic seizures with secondary generalization in adults.
Bresnahan et al. [25]	Randomized, double-blind, placebo-controlled vigabatrin trials published from Ovid 1946-October 2018 were reviewed to evaluate the efficacy and tolerability of vigabatrin as an adjunctive treatment for refractory focal epilepsy.	A 50% of greater reduction in seizure frequency may be 2-3 times more likely in patients treated with vigabatrin compared to placebo treated patients. Vigabatrin treated patients were more likely that placebo patients to have adverse effects (dizziness, fatigue, drowsiness, depression).	In people with refractory focal epilepsy, vigabatrin may significantly reduce the frequency of seizures. Adverse effects were reported at short-term follow-up.

In 2009, two parallel-group, double-blind, placebo-controlled clinical trials of 357 patients aged 18-60 years investigated the efficacy of vigabatrin as an adjunctive treatment of complex partial seizures. The first study found that out of 174 patients, 51% receiving a dose of 6 g/day and 53% receiving 3 g/day had a 50% or greater reduction in the frequency of their seizures over an eight-week baseline period and 18-week treatment period. Only 9% of patients receiving a placebo achieved a 50% or greater reduction in seizure frequency. The second study explored the median monthly frequency of complex seizures in 183 patients over an eight-week baseline period and 16-week treatment period, starting from baseline and ending at the conclusion of the study. It was found that the median monthly frequency of seizures decreased from 8.3 to 5.5 in vigabatrin-treated patients (P < 0.05) and decreased from nine to 7.5 in patients treated with placebo. Investigators determined that ~39 of vigabatrin-treated patients and 21% of placebo-treated patients had a 50% or greater reduction in seizure frequency (Table 1). The data from these two studies suggest that vigabatrin sufficiently reduced the frequency of seizures when used as an adjunct treatment of refractory complex focal seizures [2]. A study published in 2016 illustrates the five-year demographic and treatment data of adult patients in the US who are treated with vigabatrin and enrolled in the Sabril® registry - all patients treated with the drug must be registered and ophthalmic assessments documented. The study found that in 2009-2014, of ~1200 adult patients with refractory complex partial seizures, approximately 71%, 55%, and 40% of patients who had never been exposed to vigabatrin previously would remain in the registry three, six, and 12 months (Table 1). These data suggest that despite having significant resistance to AEDs and the risk of vision loss and adverse effects, adult patients continue to use vigabatrin long-term [19].

A literature review published in 2020 of randomized, double-blind, placebo-controlled trials of vigabatrin from 1946-2018 investigated the efficacy and tolerability of vigabatrin as an adjunct treatment for refractory focal epilepsy. Out of 756 patients, those treated with vigabatrin may have been two to three times more likely to achieve a 50% or greater reduction in seizure frequency when compared to those treated with a placebo. When compared with those receiving a placebo, vigabatrin-treated patients were more likely to experience dizziness, fatigue, drowsiness, and depression (Table 1). The data from this study suggest that vigabatrin treatment in patients with refractory focal epilepsy may significantly reduce the frequency of seizures and that there are some associated adverse effects. As the aforementioned studies appear to demonstrate, vigabatrin is an effective add-on treatment for refractory complex epilepsy in adults. Thus, it is important to compare its efficacy to the prevalence of vision loss, the most severe adverse effect. A 2010 systematic review of observational studies published in 1966-2009 explored the prevalence of visual field loss in patients taking vigabatrin for partial epilepsy adjunct treatment. The study found that of 1678 patients from 32 studies, 44% of those being treated with vigabatrin had visual field loss compared to 7% of the controls. In patients treated with vigabatrin, the relative risk for visual field loss was 4.0 (95% Cl 2.9-5.5). It was also found that older age and a larger dose of vigabatrin were associated with a higher proportion of visual field loss (Table 2). The data from this study suggest a significant risk of vigabatrin-associated visual field loss, necessitating that vigabatrin only be an option for refractory cases and for those whose benefit of treatment outweighs the risk [29]. When considering a thorough analysis of the efficacy of vigabatrin as an adjunctive treatment of refractory complex focal seizures, comparison to other antiepileptic drugs (AEDs) is also warranted. A 2013 meta-analysis of studies published in 1950-2009 evaluated the efficacy and tolerability of AEDs for refractory epilepsy. The study found that out of 6346 patients, three direct drug competitor trials, and 40 placebo-controlled trials, the AEDs that demonstrated the greatest short-term efficacy and tolerability were vigabatrin, sodium valproate, levetiracetam, and gabapentin. Notably, vigabatrin was associated with visual disturbances with chronic use (Table 2). These data suggest short-term efficacy and tolerability of vigabatrin, but the visual disturbances of chronic use exclude it from being considered an AED with the best balance of efficacy and tolerability [30]. Similarly, a 2018 meta-analysis of double-blind, placebo, parallel-group studies conducted between 1993 and 2013 assessed the efficacy and tolerability of 11 antiepileptic drugs (eslicarbazepine, ezogabine, gabapentin, lacosamide, levetiracetam, perampanel, pregabalin, tiagabine, topiramate, vigabatrin, and zonisamide) for add-on treatment of refractory partial-onset seizures. The study found that vigabatrin 3000 mg/day [odds ratio (OR) 6.23, 95% confidence interval (CI): 1.46-26.20] had one of the highest odds of a 50% or greater reduction in seizures, in addition to pregabalin and tiagabine. Vigabatrin also had one of the highest odds of achieving seizure freedom, in addition to ezogabine and levetiracetam. When compared to a placebo, patients were more likely to stop using any AED (except pregabalin) (Table 2). These data suggest a greater efficacy of vigabatrin treatment when compared to other AEDs and placebo and also reflect a tendency of patients to discontinue the use of any AED [31].

**Table 2:** Comparative studies

Author (Year)	Groups studied and intervention	Results and findings	Conclusions
Maguire et. al. [26]	Observational studies published from 1982-2009 that investigated the prevalence of bilateral visual field loss in patients treated with vigabatrin for refractory partial epilepsy were systematically reviewed	Of the 1678 patients identified who were treated with vigabatrin, 44% reported visual field loss compared to 7% of the controls. The relative risk for visual field loss in patients treated with vigabatrin was 4.0 (95% CI 2.9-5.5). it was also found that older age and larger dose of vigabatrin were associated with a higher proportion of patients with reported visual field loss.	There is a higher proportion of visual field loss in patients who receive vigabatrin for the treatment of refractory partial epilepsy, therefore the treatment should only be available to patients with no other options, and to patients in which the benefit of seizure reduction outweighs the risk of visual defects.
Bodalia et al. [27]	3 direct drug comparator trials and 40 placebo-controlled trials that took place from 1950-2009 were evaluated for comparative efficacy and tolerability 12 drug interventions for refractory epilepsy.	Drugs used for the treatment of refractory epilepsy that demonstrated the best short-term efficacy tolerability include vigabatrin, sodium valproate, gabapentin, and levetiracetam. Vigabatrin's association with visual changes with chronic use excludes it from joining sodium valproate, levetiracetam, and gabapentin in demonstrating the most efficient balance of efficacy and tolerability.	Vigabatrin adjunctive treatment of refractory epilepsy demonstrated short-term efficacy and tolerability but was not considered to have the best balance of efficacy and tolerability due to the visual disturbances associated with chronic use.
Slater et al. [31]	29 double-blind, placebo-controlled, parallel-group design studies conducted from 1993-2013 were analyzed to determine the efficacy and tolerability of 11 AEDs for adjunctive treatment of refractory partial onset seizures.	More than 9000 patients were more likely to have seizure freedom when treated with AEDs compared to placebo. The highest odds of 50% or greater seizure reduction were seen in patients treated with vigabatrin, pregabalin, and tiagabine. The highest odds of seizure freedom were seen in patients treated with vigabatrin, ezogabine, and levetiracetam.	When compared to placebo, eslicarbazepine, ezogabine, gabapentin, lacosamide, levetiracetam, perampanel, pregabalin, tiagabine, topiramate, and zonisamide, vigabatrin had one of the highest odds of 50% reduction in seizures and one of the highest odds of seizure freedom in patients with refractory partial-onset seizures.

Discussion

With over 7.5 million people worldwide who are affected by refractory complex partial seizures, epilepsy remains a highly studied medical phenomenon [32, 33]. First-line treatment remains medical management with roughly 50% of those diagnosed with epilepsy to control their side effects; it is still a medical journey to get that number as close to 100% as possible. 

First-line agents for refractory complex partial seizures include but are not limited to valproic acid, topiramate, zonisamide, and lamotrigine [34]. While some seizures are controlled with monotherapy, many are controlled with polytherapy. Vigabatrin has been proven across many clinical trials to be generally a safe drug. Many of those double-blind random studies indicate that vigabatrin sufficiently reduced the frequency of seizures when used as an adjunct treatment of refractory complex focal seizures [2]. The major side effects of headache and dizziness were not severe enough to discontinue the medication in any trial [35]. Nearly 30% of patients did experience peripheral visual defects within five years, with many of these reversible with medication cessation. Teratogenicity is not fully understood but has shown to be detrimental in preclinical mice trials, indicating it is not currently safe to trial in a pregnant human population [27].

The efficacy along with the potential for side effects should be discussed between the patient and the physician. Factors that should determine treatment should include number of seizures, duration of seizures, occupation, and lifestyle. If the physician believes that the benefits outweigh the risks in a non-pregnant person, specifically peripheral vision loss, the patient should be aware of this black box warning and ultimately be able to make the decision. Patients should also be made aware that older age may put them at an increased risk for peripheral visual defects [26]. However, it is important to carefully monitor patients for any adverse effects, particularly long-term use of the drug, and to discontinue the drug if serious side effects occur.

Antiseizure medications will continue to be studied as more people are diagnosed with epilepsy around the world [36]. Vigabatrin studies may help us discover the avoidance of detrimental side effects or deaths. Future studies may include vigabatrin efficacy in different demographics along with the efficacy of Vigabatrin in combination with specific seizure medication versus others [37-40]. Future studies may conduct a meta-analysis to complement and reinforce these findings.

## Conclusions

Sabril is an antiepileptic medication prescribed as an additional treatment for refractory complex partial seizures in patients at least ten years old who have not responded well to other alternative therapies. Multiple clinical trials indicate that Sabril sufficiently reduces the frequency of seizures when used as an adjunct treatment of refractory complex focal seizures. However, it is important to carefully monitor patients for any adverse effects, particularly long-term use, and to discontinue the drug if serious side effects occur.
